# Thermal Efficiency Analysis for Laser-Assisted Plasma Arc Welding of AISI 304 Stainless Steel

**DOI:** 10.3390/ma12091460

**Published:** 2019-05-06

**Authors:** Dominik Hipp, Achim Mahrle, Eckhard Beyer, Sebastian Jäckel, Martin Hertel, Uwe Füssel

**Affiliations:** 1 Institute of Manufacturing Science and Engineering, TU Dresden, P.O. Box, D-01062 Dresden, Germany; achim.mahrle@iws.fraunhofer.de (A.M.); eckhard.beyer@tu-dresden.de (E.B.); sebastian.jaeckel@tu-dresden.de (S.J.); martin.hertel@tu-dresden.de (M.H.); uwe.fuessel@tu-dresden.de (U.F.); 2 Fraunhofer IWS Dresden, Winterbergstraße 28, D-01277 Dresden, Germany

**Keywords:** hybrid welding, laser welding, plasma arc welding, process efficiency, interaction mechanisms, synergistic effects

## Abstract

Synergistic effects during hybrid laser-arc welding may cause increased process efficiencies. However, the basic interactions behind these effects are still being discussed, with some contradictory reports. In this study, particular welding parameters of interest were systematically varied to further the understanding of involved phenomena. The experimental trials are evaluated regarding their synergistic achievements in terms of process efficiency, melting efficiency and energy coupling efficiency using a factorial two-level Design-of-Experiment (DoE) approach. The results show that the growth in process efficiency can be attributed to a dramatic increase in melting efficiency whereas the energy coupling efficiency is only moderately increased. Thus, the synergistic effect is mainly caused by secondary mechanisms that change the energy usage inside the workpiece while direct interactions between the two heat sources can be excluded as a reasonable cause for increased process efficiencies. It is concluded that the different sizes of the heat sources change the heat and mass flow positively and consequently lead to a higher performance level.

## 1. Introduction

Hybrid laser-arc welding techniques have gained further importance in the recent years. However, the lack of knowledge about the basic interactions hinders the development of new technologies and the further optimization of existing ones. Thus, it is of utmost importance to study and clarify the involved effects. During laser-assisted arc welding two effects are of particular interest. Fuerschbach [1] observed a focusing of the plasma arc in the presence of a laser beam while Mahrle et al. [2] demonstrated a stabilization of a plasma arc at high welding speeds and a less fluctuating arc voltage. Therefore the first effect refers to a more stable processing regime. Second, the weld performance of hybrid processes is notably increased compared to conventional processes. Both, Fuerschbach [3] and Mahrle et al. [4] achieved deeper and wider fusion zones through the combination of a laser beam and a plasma arc, Hu and den Ouden [5] noted a higher melting efficiency during hybrid welding and Liu et al. [6] observed a melting efficiency of more than 50% dependent of the positioning of and the distance between the two heat sources. Steen [7], who first combined a laser beam and a welding arc with the aim of augmenting the performance of a low power CO_2_-laser beam, has already reported synergistic effects for the combination of the two heat sources. He found that the laser beam is able to stabilize the Tungsten Inert Gas (TIG) arc and that the feed rate for full penetration welds can be increased. These results stimulated a lot of experimental and theoretical work aimed at an explanation of these effects. Based on calculations, Paulini and Simon [8] concluded that these achievements can result from an increased concentration of metal vapor escaping from the laser induced keyhole into the arc region. Due to their lower ionization potential the metal atoms were thought to be responsible for a decrease of the electrical resistance of the arc discharge path and thus an increase of the power density and the stability of the arc. This argumentation seemed to be reasonable and was later taken over by several other researchers [9,10,11,12]. The studies of Chen et al. [13] and Liu and Chen [10], both showing a higher metal atom concentration during the combined process compared to the conventional arc welding, were interpreted as an evidence for this hypothesis. However, the experiments of Fuerschbach [1] showed, that synergistic effects are only achievable under certain process conditions and that some process regimes do not show any synergistic effects during the combination of the two heat sources. Mahrle et al. [14] further revealed that the stabilization effect for their applied setup was more pronounced at lower laser intensities—where a keyhole formation and an extensive metal vapor evaporation is unlikely—and concluded that the metal vapor can only play a secondary role for arc root and arc column stabilization. The numerical calculations of Schnick et al. [15] revealed a decreasing arc core temperature in regions with high metal vapor concentrations due to increased radiation losses, which was interpreted as a disproof of the chain of reasoning of the metal vapor theory. Mahrle et al. [16] then hypothesized that the preheating of the material by the welding arc can be considered as another possible reason for the synergistic effect. It was proposed that a possible initiation of a keyhole at low laser intensities is favored because the laser beam already strikes pre-heated molten material whereby heat conduction losses into the surrounding area of the laser spot are effectively reduced. Stute et al. [17] suggested another hypothesis and explained synergistic effects in laser-arc processing as a result of the optogalvanic effect. They stated that the laser radiation is partially absorbed by the arc plasma giving rise to increased arc temperatures, a higher conductivity and thus a higher melting capability [18].

All previously mentioned explanations of synergistic effects in hybrid laser-arc processing imply an increase in net energy transfer from the heat sources to the material. Consequently, as a result of those effects, pronounced improvements of the process efficiency should be caused by an increased energy coupling efficiency of those processes. In this context the thermal efficiency or overall process efficiency *η_T_* corresponds to the ratio of power *P_U_* which is needed to melt the weld material per unit time (without losses) to the total applied power *P_A_*. This quantity can be split according to Equation (1) in the melting efficiency *η_M_* (energy usage inside the base material) and the energy coupling efficiency *η_C_* (energy input from the heat sources) by using the power *P_T_* which is transferred from the heat sources to the workpiece [19].

(1) $$\eta_{T}=\frac{P_{U}}{P_{A}}=\eta_{M}\times \;\eta_{C}=\frac{P_{U}}{P_{T}}\;\times \;\frac{P_{T}}{P_{A}}$$

Contrarily, some researchers also stated a more efficient energy usage inside the workpiece and therefore a higher melting capability as a reason for the increased performance level in laser-arc processing. Corresponding mechanisms should be referred to secondary effects since they are not related to direct interactions between laser radiation and arc plasma. For example, Matsuda and Utsumi [20] proposed that a surface depression occurs under the action of the arc pressure, which Beyer et al. [21] used to formulate the hypothesis of a possible reduction of the effective sheet thickness leading to an increase in process performance. In addition, the consideration of the characteristic sizes of the two heat sources can provide other arguments. Since the size of the welding arc root typically exceeds the size of the laser beam spot by more than one order of magnitude, heat conduction losses from the laser-irradiated area to the base material are reduced. Therefore the strongly focused energy of the laser can be more efficiently used to melt the material under the conditions of the superposition of both heat sources.

It is concluded that a carefully conducted experimental efficiency analysis of laser-arc processes will allow for a more profound discussion of the most vital interaction mechanisms. Hu and den Ouden [5] compared in their study the energy coupling efficiency and the melting efficiency of a laser beam and a TIG welding arc to the performance of the combined laser-TIG process. The results indicate, that the energy coupling efficiency of the combined process remains on the same level while the melting efficiency drastically increases compared to the separate processes. However, only dependencies on the welding current as the main welding parameter were studied. As pointed out before, the occurrence of synergistic effects might strongly depend on process parameters [1,14] wherefore further experiments are needed to allow a valid interpretation of the results. This study uses a factorial Design-of-Experiments (DoE) approach with consideration of four factors to systematically evaluate the influence of the secondary welding parameters laser power, laser beam radius, plasma gas flow and working distance during laser-assisted plasma arc welding (LaPAW) on the process efficiency. In a DoE approach the input factors are systematically varied and the effect on the target values defined beforehand is quantitatively estimated. Therefore it is possible to identify individual significant factors and furthermore, the interactions among them. Thus, it is the perfect tool to identify parameter sets with the highest efficiency. Through the evaluation of the dependence of the efficiency values on process parameters, the clarification of the synergistic effects during LaPAW can be further approached with the results of the DoE.

The results of this study will contribute to the question for the unresolved physical explanation of the synergistic effects occurring during hybrid laser-arc welding. The statistical efficiency analysis will hopefully shed light on selected hypotheses postulated in literature. This will result in a more profound process understanding and can be applied for further process developments and process performance increases.

It should be noted that the considered process of LaPAW differs in many aspects from common hybrid laser-arc welding [22,23]. Current applications of hybrid laser-arc welding mostly involve setups with different interacting zones of laser beam and electrical arc as well as consumable electrodes [24,25,26,27,28], while LaPAW is conducted in a coaxial arrangement with a non-consumable tungsten electrode.

## 2. Materials and Methods

**Figure 1:**
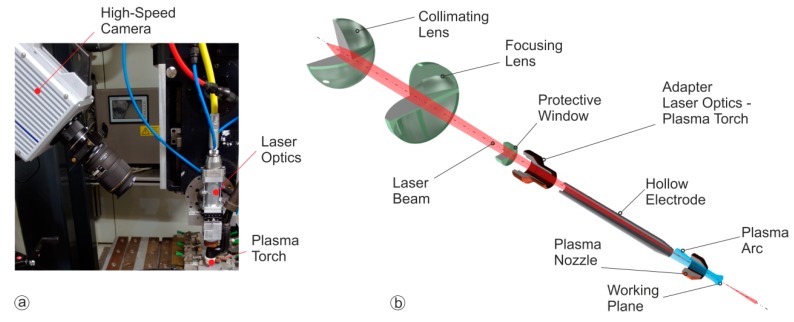
Experimental setup of the Laser assisted Plasma Arc Welding (**a**) and internal embodiment (**b**).

For the welding experiments a plasma torch with a non-consumable hollow tungsten cathode was combined with a low-power laser beam optic (Figure 1). With this setup the laser beam and the plasma arc are operating in the same processing zone in a coaxial arrangement.

A single mode fiber laser with a wavelength of 1.07 µm and a maximum output power of 600 W was used as a beam source. The laser beam was guided through the hollow electrode using a collimating and focusing lens and resulting in a laser beam radius ω_0_ (1/e^2^) of between 50 and 100 µm. To generate the plasma arc a hollow tungsten electrode was used. The arc operated in the Direct Current Electrode Negative (DCEN) mode. The partitioning of the plasma torch and the laser beam optics was realized through a particularly constructed adapter which isolates the two components from each other (thermally and electrically) and closes the plasma chamber with the inserted protective window.

The process window was determined in preliminary test series to cover a maximum area of settings while still ensuring a stable welding process for the combined as well as the separate processes. The main welding parameters, welding current and welding speed, were kept constant at 120 A and 0.4 m/min to ensure nearly comparable levels of linear welding energy for all performed welding trials. Pure Argon was used as plasma gas as well as shielding gas (flow rate = 10 L/min). The exit diameter of the plasma nozzle amounted to 3 mm. For the DoE approach the commercially available software Design Experts 10 was used and a two-level full factorial design with four factors and one center point was chosen. Since the effect of the welding current as the main factor influencing process outcome of LaPAW was already studied in literature [5], in this study the varied factors were the remaining important parameters of LaPAW namely **(A)** Laser beam radius ω_0_, **(B)** Laser power P_L_, **(C)** Plasma gas flow rate Q_P_ and **(D)** Working distance d_w_ between the plasma nozzle and the workpiece. The evaluated factor levels are given in Table 1, the individual combination of process parameters can be found in Table A1 in the Appendix A. The experimental design consisted of 2^4^ = 16 principal runs. Additionally, the center point (level 0) was repeated five times for a better estimation of the variance.

The method for determining the energy coupling, melting and thermal efficiency is described in detail by Hipp et al. [19] and consists of a contactless thermographic measurement of surface temperatures inside defined measuring areas during the process and the cooling regime. Instead of measuring the maximum temperature inside the molten pool, which is highly sensitive to errors [29], this technique measures the temperatures in areas located outside the processing zone. The resulting time-dependent temperature profiles inside the measuring areas are then aligned with a corresponding heat flow computation model. Using these data, the heat flux to the workpiece and therefore the energy coupling efficiency *η*_c_ can be inversely computed. Then, for the determination of the thermal efficiency value *η_T_*, the evaluated weld seam cross sections are used in combination with Equation (2) [19]:

(2) $$\eta_{T}=\;\frac{P_{U}}{P_{A}}=\frac{v_{x}\cdot A_{S}\cdot \rho \cdot \left(c_{p}\cdot \left(\vartheta_{s}-\vartheta_{\infty }\right){\;+\;h}_{s}\right)}{U_{\mathrm{Arc}}\cdot I_{\mathrm{Arc}}{+P}_{L}}$$

where *v_x_* is the welding speed, *A_S_* the weld seam area, *ρ* the density of the probe, *c_p_* the specific heat capacity, *ϑ_S_* and *ϑ_∞_* the melting and the ambient temperature, *h_s_* the enthalpy of fusion, *P_L_* the laser power, *U_Arc_* and *I_Arc_* the arc voltage and current, respectively. The melting efficiency *η_M_* then results from applying Equation (1). The efficiency determination method and the used computational model were evaluated regarding their accuracy by Hipp et al. [19].

The efficiency values were used as dependent variables (responses) for the statistical analysis. The arc voltage was recorded during the weld trials for the proper determination of the overall arc input power and considered as a dependent variable. All runs were conducted on 200 × 60 × 3 mm^3^ AISI 304 (X5CrNi18-10) stainless steel sheets. After the experiments, the weld specimen were cut, ground, polished and etched with Adler solution to measure the weld cross section area using optical microscopy. During all experiments the molten pool was observed using a high speed camera (see Figure 1). The camera was inclined to visualize a possible formation of a surface depression or a laser keyhole.

## 3. Results and Discussion

**Figure 2:**
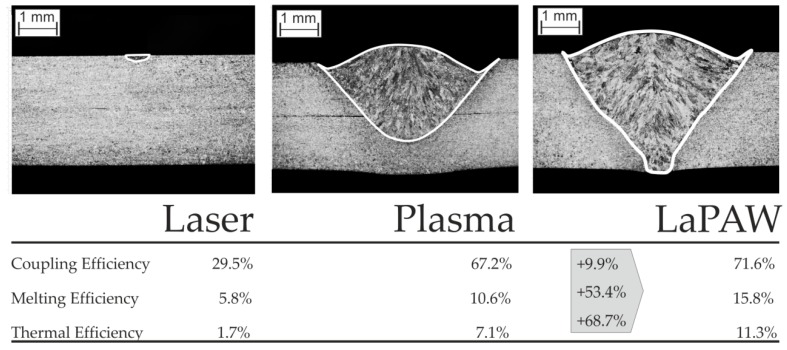
Cross sections for laser beam welding (P_L_ = 200 W; ω_0_ = 200 µm), plasma arc welding (Q_P_ = 1.8 L/min; d_W_ = 5 mm) and the laser-assisted plasma arc welding (LaPAW) process (P_L_ = 200 W; ω_0_ = 200 µm; Q_P_ = 1.8 L/min; d_W_ = 5 mm) with corresponding efficiency values.

**Figure 3:**
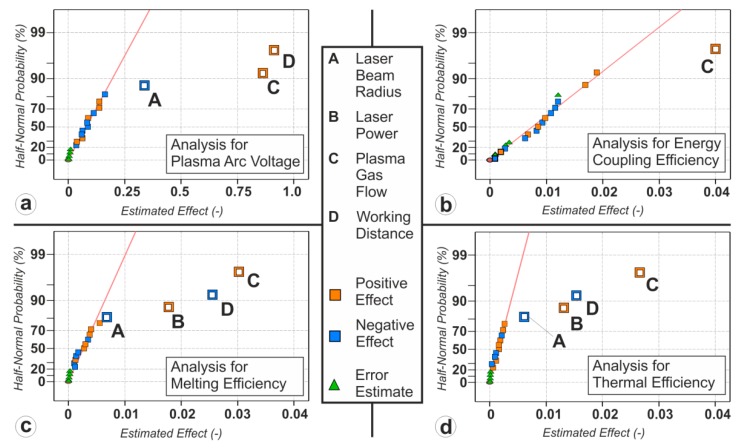
Results of the statistical analysis for the plasma arc voltage (**a**), energy coupling efficiency (**b**), melting efficiency (**c**) and thermal efficiency (**d**).

The efficiency values were evaluated as described before under the usage of Equation (2), the results of a computational model and the weld seam cross sections. The results of all weld trials can be found in Table A1 in the Appendix A. Although the cross sections are in this context only an expedient for the efficiency analysis, the interpretation of them is of special interest. Therefore in Figure 2 the characteristic weld seam cross sections of laser beam and plasma arc welding trials as well as of the combined LaPAW process are presented. While the laser beam with a power of 200 W and a focus spot diameter of 200 µm barely melts the material, the plasma arc welding process with an arc power of about 2 kW achieves a weld penetration of about 2/3 of the workpiece thickness for the applied parameter constellation. The combination of both processes produces a full penetration weld. The corresponding efficiency values indicated in Figure 3 and Table A1 confirm the results of Hu and den Ouden [5]. Whereas the coupling efficiency is only moderately increased by about 10% in comparison to the arithmetical coupling efficiency of the individual processes, the melting efficiency of the combined process is about 1.5 times higher than the melting efficiency of the pure plasma arc process. This finding obviously indicates that the increased weld cross section area must be ascribed to the more beneficial usage of energy inside the probe. These results are in line with the results of an efficiency analysis with 1 mm standard type 304 stainless steel presented by Hipp et al. [19], despite the increase being less pronounced. This leads to the assumption that the heat flow inside the probe—driven either by conductive and/or convective transfer mechanisms—is beneficially changed to generate the resultant weld seam cross-section with increased penetration by more favorable thermal and/or fluiddynamical boundary conditions. This is further considered as clear evidence for the hypothesis that secondary, i.e., thermal, effects are responsible for synergistic performance benefits in laser-arc processing.

The measured values of the energy transfer efficiency are consistent with values reported in literature. The laser beam welding process obviously operates in the heat conduction mode where multiple reflections are absent and the energy transfer efficiency is in the range of the averaged absorptivity of the material of A = 27% [30]. The coupling efficiency of plasma arc welding is high and outreaches some reported values of *η_c_* = 47 ± 3% by DuPont and Marder [31]. However, Evans et al. [32] stated that the plasma arc efficiency strongly correlates to the particular process setup and reported achievable energy coupling efficiencies in the range of 50–67%. Also the determined melting efficiencies of the LaPAW are comparable to literature values, for example, from Hu and den Ouden [5].

The results of the statistical analysis for the evaluated process parameters are illustrated in Figure 3 as a half-normal probability plot. The abscissa presents the estimated effect in terms of absolute values. If there is no correlation between the dependent variable and the factors (i.e., the varied process parameters), the estimated effect is zero. Under the assumption that all observed effects are normally distributed, the half-normal probability of one factor equals the area under this normal distribution up to the estimated effect of this factor. This is presented on the ordinate of the plot. Thus the half-normal probability plot can perfectly serve as a visual tool to identify the significant parameters since their estimated effect is greater than zero and the half-normal probability is high, wherefore they appear in the far right top in the diagram. Factors that do not have any influence on the dependent variable appear close to zero located on a line. In this study only effects with a significance threshold of a = 0.01 in the corresponding Analysis of Variance (ANOVA) are selected as significant.

### 3.1. Arc Voltage

**Figure 4:**
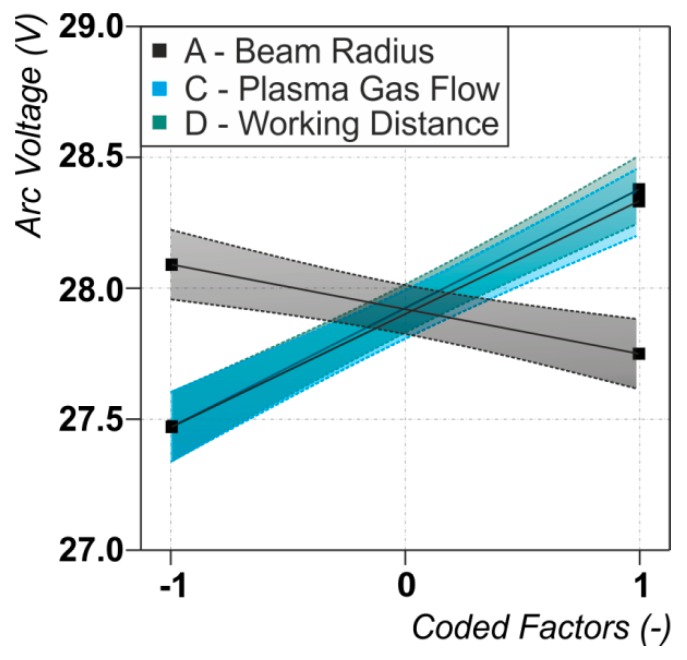
Dependencies between the arc voltage and the significant process parameters. For the values of the varied process parameters (coded factors) see Table 1.

The half-normal probability plot of the plasma arc voltage (Figure 3a) as well as the graph showing the dependences between the process parameters and the arc voltage (Figure 4) reveal that the voltage strongly depends on the plasma gas flow rate and the working distance (both positive) while there is a weak dependence on the laser beam radius (negative). During the experiments a mean value of the arc voltage of U_ARC,mean_ = 27.8 V with a standard deviation of σ = 0.6 was observed. The applied linear model in the ANOVA fits well with an R^2^ = 0.933 and an adjusted R^2^ = 0.92. Therefore, more than 90% of the observed effects can be explained by the applied model, leading to reliable conclusions.

The observed dependencies of the arc voltage on working distance, i.e., arc length, and plasma gas flow rate are widely validated in literature [33]. More complex is the interpretation of the change of arc voltage with the laser beam radius. The negative sign of this effect indicates that greater spot diameters lower the arc voltage. Mahrle et al. [16] already extensively studied the influence of the laser beam size and location on the arc voltage. The study revealed the main reason for a measured drop in arc voltage to be a positioning effect of a displaced arc root spot back to the coaxial axis of the torch by the laser beam. Therefore the effective arc length is shortened causing a smaller arc voltage. The rise of the arc voltage by smaller laser beam radii can be a consequence of the already mentioned cooling effect of metal vapor on the arc plasma [15]. Since the intensity of the laser beam is indirectly proportional to the squared spot size diameter, those effects are more pronounced at smaller spot sizes and—as the case may be—higher evaporation rates.

### 3.2. Energy Coupling Efficiency

**Figure 5:**
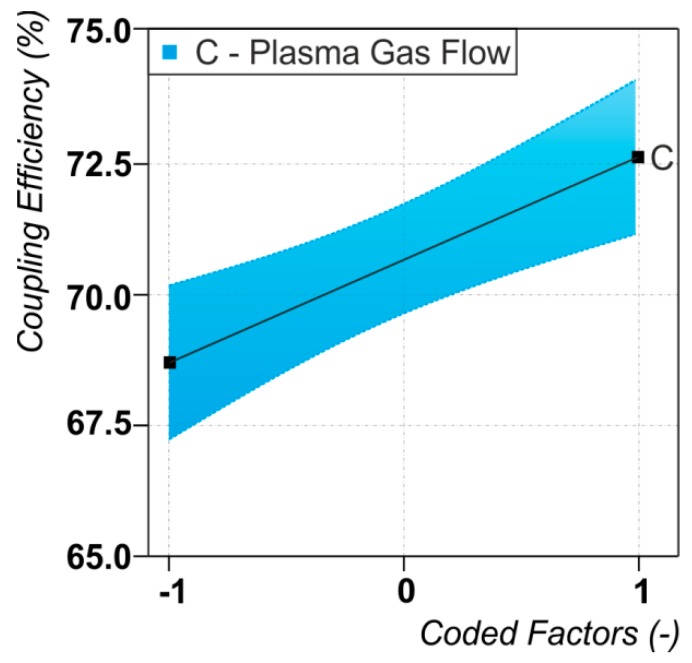
Dependencies between the energy coupling efficiency and the significant process parameter. For the values of the varied process parameters (coded factors) see Table 1.

The results of the statistical analysis for the dependence of the energy coupling efficiency are presented in Figure 3b, the dependencies between the energy coupling efficiency and the significant process parameter are shown in Figure 5. Only the plasma gas flow rate (C) is identified as a significant factor. The mean of the absolute value of the energy coupling efficiency in the studied process parameter range is η_C,Mean_ = 70.1% with a standard deviation of σ = 2.7%. The highest measured value is η_C,Max_ = 74.1% and the lowest η_C,Min_ = 65.3%. One should mention here, that the fit of the linear model through the ANOVA is pretty poor resulting in an R^2^ = 0.47 and an adjusted R^2^_adj_ = 0.44. In other words, only about 50% of the observed effects can be explained by the applied linear regression model. The test of curvature is insignificant, wherefore quadratic dependences can be excluded. This indicates that the dependency of the energy coupling efficiency on the plasma gas flow might be of a higher order. However, due to the fact that the overall thermal efficiency is much more affected by the melting capability, detailed investigations of those relationships were not further pursued in this study. Moreover, since the factor plasma gas flow is found to be significant to a significance level of α = 0.01, the statements concerning this dependency and the consequent indications still hold true even for a low model quality.

Concerning relationships between dependent variables (responses) it is found that the energy coupling efficiency correlates to the arc voltage (correlation coefficient = 0.7) which in turn was dependent on working distance, plasma gas flow rate and beam radius. This finding indicates that the increase in arc voltage with arc length (working distance) and the corresponding increase in arc power must be compensated by higher energy losses through the increased lateral surface of the arc to the environment and consequently does not contribute to a higher amount of transferred energy to the workpiece. Additionally, the discussed probability of higher evaporation rates with a more intense laser beam does also increase the losses (as metal vapor), and consequently shows no influence on the energy coupling efficiency.

It is worthwhile to emphasize that the energy coupling efficiency does not depend on any of the studied laser parameters. This is considered as an indication that direct interactions between laser radiation and arc plasma—whether they are present or not under the conditions of the performed study—are not capable of improving the energy coupling efficiency of the process. In other words, it can be stated that direct interactions between laser radiation and arc plasma are not a stringent necessity for the synergistic effect of increased process efficiency.

### 3.3. Melting Efficiency

**Figure 6:**
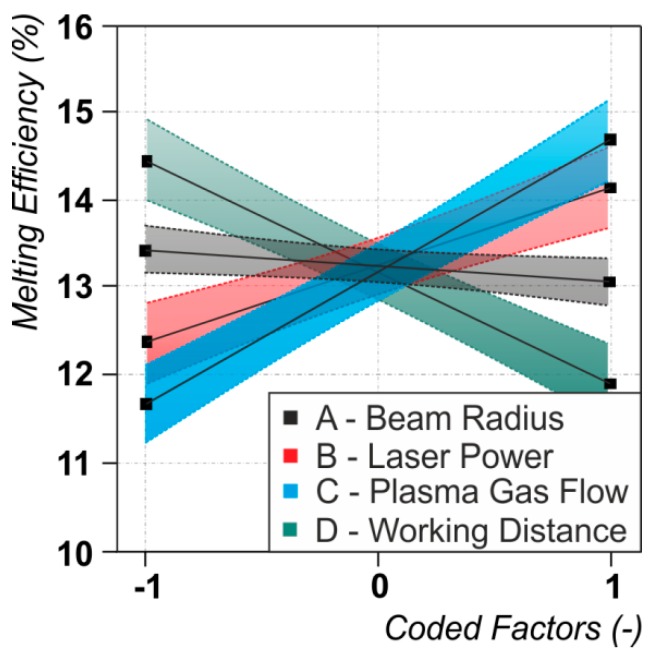
Dependencies between the melting efficiency and the significant process parameters. For the values of the varied process parameters (coded factors) see Table 1.

As can be seen in Figure 3c and in Figure 6, vital factors for the melting efficiency are the plasma gas flow (C, positive), the working distance (D, negative) and the laser power (B, positive), while the laser beam radius (A, negative) only plays a minor part. Hence, a higher plasma gas flow rate and a higher laser power offer beneficial conditions for the energy usage inside the workpiece while a higher working distance decreases it. The laser beam radius is not discussed at this point, since the related effect is negligible. The mean value in the descriptive statistical analysis of the melting efficiency is η_M,Mean_ = 13.9% with a standard deviation of σ = 2.3%. The highest value of the observed melting efficiency is η_M,Max_ = 18.4% and the lowest is η_M,Min_ = 10.1%. The fit of the data through the ANOVA with an R^2^ = 92.8 and an adjusted R^2^_adj_ = 91.5 is good, leading to reliable conclusions about the impact of the investigated factors.

**Figure 7:**
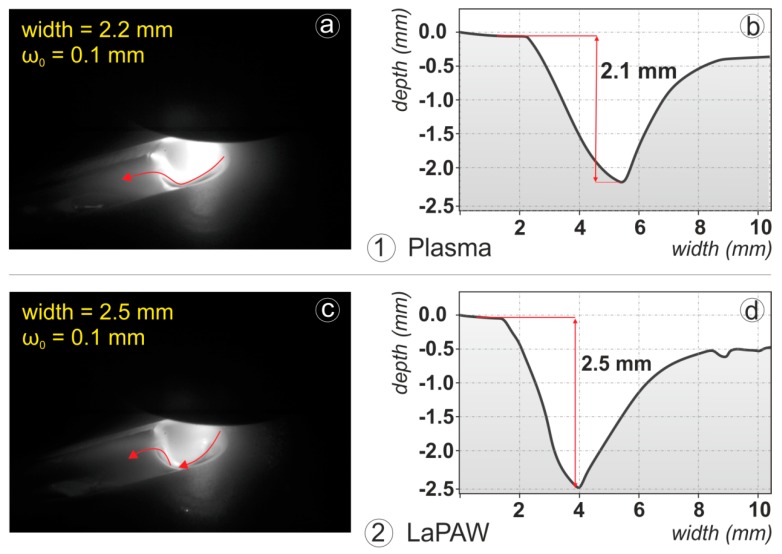
High speed images of the molten pool (**a**,**c**) and the corresponding depth of the solidified crater (**b**,**d**) at process end for conventional plasma arc welding (**1**) and Laser assisted Plasma Arc Welding (**2**).

The highest efficiency was observed at high plasma gas flows and low working distances. In combination these factors obviously lead to a higher arc pressure on the surface of the molten pool. The high speed image in Figure 7a reveals, that this causes a pronounced depression of the molten pool surface of the conventional plasma welding process. Through the measurement of the solidified end crater size of the welding process (see Figure 7b) it is supposed, that this cavity is about 2 mm in depth (From the High Speed Images of the process end, it follows that the real depth should be deeper than the measured solidified craters. A re-flux of molten material into the crater was observed). The laser beam spot then hits the ground of this cavity during LaPAW and the effective workpiece thickness, which the laser has to fuse for achieving a full penetration weld, is drastically reduced to only 1 mm. Therefore the energy of the laser is supposed to be used more effectively to melt the remaining material with increased overall melting efficiency.

An explanation for the significance of the laser power can be as follows. The laser hits the surface of the workpiece which is already at melting temperature. In this case the whole energy of the laser is available to melt additional material [16] and also the molten pool characteristics are changed. This is clearly demonstrated in Figure 7c,d. The high speed image reveals a more deepened cavity of the LaPAW process compared to the conventional plasma welding (Figure 7c). Interestingly, the built up cavity is in the size of the plasma arc, while a laser keyhole is evidently not observable. It is believed that the further energy of the laser beam causes higher temperatures of the molten material and thus reduces its viscosity and its surface tension. This affects the dynamics of the melt pool heat and mass flow positively, resulting in an increased melting efficiency. Another possible effect is the suppression of heat conduction losses from the center of the molten pool—where the laser hits the material—to the surrounding base material through the characteristic size of several millimeters of the plasma arc. The heat conduction then primarily takes places in one direction, namely in line with the workpiece thickness.

### 3.4. Thermal Efficiency

**Figure 8:**
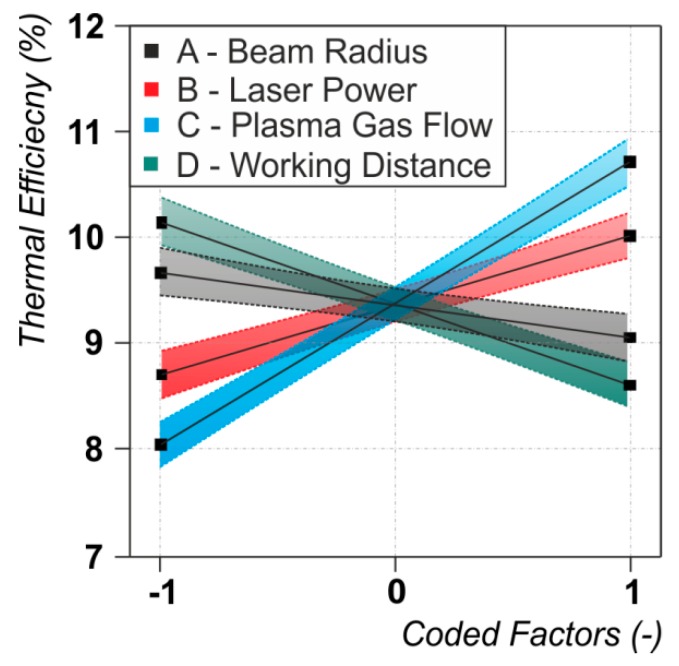
Dependencies between the thermal efficiency and the significant process parameters. For the values of the varied process parameters (coded factors) see Table 1.

For the observed process parameter range the thermal or overall process efficiency of the LaPAW process strongly depends on the plasma gas flow (C, positive) and moderately on the working distance (D, negative), the laser power (B, positive) and the laser beam radius (A, negative) (see Figure 3d and Figure 8). As is the case for the melting efficiency, all studied process parameters have an influence on the thermal efficiency. Since the thermal efficiency is directly coupled with the other efficiency values through Equation (1) it is not surprising that all the factors which have an influence on either the energy coupling efficiency (C) or melting efficiency (C, D, B, A) are significant. Independent of the acting physical phenomena, it can be stated at this point, that for achieving high process efficiencies, high plasma gas flow rates and low working distances are needed. Furthermore, although only subsidiary welding parameters were analyzed, the estimated thermal efficiency lies in the range of 7 and 13.3%. Thus, almost a doubling of the thermal efficiency between the least and most beneficial configurations was observed.

## 4. Summary and Conclusions

The present study tried to address the still unresolved questions about the phenomena occurring during laser-arc hybrid welding leading to synergistic effects. Therefore the energy coupling, melting efficiency and thermal efficiency were measured in dependence on the process parameters spot diameter, laser power, plasma gas flow and working distance. The results show, compared to the individual plasma arc welding process, a small increase in energy coupling efficiency but a considerable increase in melting efficiency of the LaPAW process. It can be concluded that no direct interactions between the two heat sources enhance the energy transfer into the workpiece. Instead, the improved process performance is a result of a more efficient energy usage inside the molten pool. The significance of the laser power in the analysis of the melting efficiency demonstrates that a positively changed heat and mass flow inside the material is capable of producing the mentioned synergistic effects. Furthermore, it is concluded that because of the significant correlation between the melting efficiency and the plasma gas flow (positive) as well as the working distance (negative), a strong surface depression occurs. The high speed images reveal a surface deformation of about 2 mm in depth. With this knowledge, further process optimizations should concentrate on the improvement of heat and mass flow inside the workpiece.

## Figures and Tables

**Table 1:** Process parameters for level “−1” and “1” as well as additional center points (level 0) in the Design of Experiments approach.

Level:	−1	1	0
**(A)** Laser beam radius ω_0_ (µm)	50	100	75
**(B)** Laser power P_L_ (W)	100	200	150
**(C)** Plasma gas flow Q_P_ (L/min)	1.2	1.8	1.5
**(D)** Working distance d_w_ (mm)	3	5	4

## Appendix A

**Table A1:** Results of the experiments for the statistical analysis as well as the corresponding trials with laser and plasma only.

A-Beam Radius (µm)	B-Laser Power (W)	C-Plasma Gas Flow (L/min)	D-Working Distance (mm)	Weld Seam Cross Section (mm^2^)	Arc Voltage (V)	Coupling Efficiency	Melting Efficiency	Thermal Efficiency
200	100	1.8	5	5.87	28.5	0.686	0.137	0.094
200	200	1.8	3	7.88	27.6	0.686	0.184	0.126
200	200	1.8	5	7.28	28.5	0.716	0.158	0.113
100	200	1.2	3	6.46	27.1	0.667	0.157	0.105
200	100	1.8	3	6.84	27.7	0.733	0.153	0.112
100	200	1.8	3	8.50	28.2	0.726	0.183	0.133
100	100	1.2	5	4.80	28.1	0.665	0.117	0.078
100	200	1.2	5	5.38	28.1	0.703	0.119	0.084
100	100	1.8	5	6.62	28.5	0.741	0.142	0.105
200	100	1.2	3	4.83	26.8	0.653	0.126	0.082
100	100	1.2	3	5.70	26.9	0.665	0.144	0.096
200	100	1.2	5	4.31	27.8	0.692	0.101	0.070
100	100	1.8	3	7.01	28.0	0.705	0.162	0.114
200	200	1.2	3	5.90	26.9	0.665	0.146	0.097
200	200	1.2	5	5.32	27.7	0.699	0.122	0.085
100	200	1.8	5	7.46	29.3	0.731	0.155	0.113
**Laser only**								
100	100	-	-	0.04	-	0.250	0.088	0.022
100	200	-	-	0.17	-	0.310	0.155	0.048
200	200	-	-	0.06	-	0.295	0.058	0.017
200	100	-	-	0.02	-	0.292	0.038	0.011
**Plasma only**								
-	-	1.2	3	4.91	27.2	0.631	0.133	0.084
-	-	1.8	3	6.00	28.4	0.717	0.138	0.099
-	-	1.8	5	4.46	29.5	0.672	0.106	0.071
-	-	1.2	5	3.38	28.2	0.558	0.100	0.056
**Center point**								
150	150	1.5	4	5.38	27.9	0.699	0.123	0.086
150	150	1.5	4	5.36	27.9	0.697	0.123	0.086
150	150	1.5	4	5.38	28.0	0.723	0.119	0.086
150	150	1.5	4	5.55	28.0	0.731	0.122	0.089
150	150	1.5	4	5.48	28.1	0.735	0.118	0.087
**Fixed parameters**								
Welding Speed: 0.4 m/min; Diameter plasma nozzle: 3 mm; Welding current: 120 A; Flow rate of shielding gas; 10 L/min								
